# Supplementary material for: Dietary circadian rhythms and cardiovascular disease risk in the prospective NutriNet-Santé cohort
Source: Nat Commun. 2023 Dec 14;14:7899. doi: 10.1038/s41467-023-43444-3 (PMC10721609; doi:10.1038/s41467-023-43444-3)
Supplement: Supplementary file 1 — Supplementary Information [file 41467_2023_43444_MOESM1_ESM.pdf]

Supplementary method A. Identification of energy under-reporting

Energy under-reporting was identified using Black's method [38,86] based on the original method developed by Goldberg et al. [87], relying on the hypothesis that energy expenditure and intake, when weight is stable, are equal. Black's equations are based on an estimate of the person's basal metabolic rate (BMR) calculated via Schofield's equations [88] and taking into account sex, age, height and weight, as well as physical activity level (PAL), number of 24h records, intra-individual variabilities of reported energy intake and BMR, and intra/intervariabilities of PAL. In the present study, intra-individual coefficients of variations for BMR and PAL were fixed using the values proposed by Black et al., i.e. 8.5 % and 15%, respectively. For identifying under-reporters, the 1.55 value of PAL was used. It corresponds to the WHO value for "light" activity, which is the probable minimum energy requirement for a normally active but sedentary individual (not sick, disabled or frail elderly). A higher value might have exaggerated the extent of under-reporting. Some under-reporting individuals were not excluded if their reported energy intake, initially estimated abnormally low, was found to be likely in case of recent weight variation or reported practice of weight-loss restrictive diet or proactive statement of the participant that he/she ate less than usual on the day of the dietary record. In this study 18,818 participants (corresponding to 14.7% of the subjects) were considered as under-energy reporters and were excluded from the study. This proportion of under-reporters is common in France, for instance in the nationally representative INCA 3 study conducted in 2016 by the French Food Safety Agency [89] 18% of adults participants were identified as under-reporters using the Black method.

Supplementary method B. Deriving dietary patterns by principal component analysis and corresponding factor loadings

The healthy and the western dietary patterns were obtained by a principal-component analysis (PCA) that was built on the basis of 20 predefined food groups and was produced using the SAS "Proc Factor" (SAS Institute Inc., Cary, North Carolina). The PCA combines food groups in linear models, grouping correlated variables. The correlation coefficients among variables are named factor loadings and can be positive, for positive correlation with the factor, or negative, for inverse association with the factor. In this analysis we considered food groups with a factor loading under -0.25 or over 0.25. Then we used the SAS "Varimax" option to rotate factors by orthogonal transformation and to maximize the independence (orthogonality) of retained factors and obtain a simpler structure for easier interpretation. We considered eigenvalues greater than 1.25, the scree test (with values being retained at the break point between components with large eigenvalues and those with small eigenvalues on the scree plot), and the interpretability of the factors to determine the number of factors to retain. For each participant, we obtained the factor score for each pattern by summing observed consumption from all food groups, weighted by the food group factor loadings. Overall, the obtained factor score represents the conformity between the diet of an individual and the given pattern. Labelling was descriptive, based on foods most strongly associated with the dietary patterns. The healthy pattern (explaining 10.6% of the variance) was characterized by higher intakes of fruit, vegetables, soups and broths, unsweetened soft drinks and whole grains and lower sweetened soft drinks intake. The Western pattern (explaining 7.0% of the variance) was characterized by higher intakes of fat and sauces, alcohol, meat and starchy foods.

	Factor loadings for the Healthy Pattern	Factor loadings for the Western Pattern
Alcoholic drinks	-.09	**0.28**
Breakfast cereals	0.07	-.18
Cakes and biscuits	-.19	0.00
Dairy products	0.06	-.01
Eggs	0.07	0.04
Fats and sauces	0.01	**0.54**
Fish and seafood	0.20	0.10
Fruit	**0.35**	0.05
Meat	-.18	**0.31**
Pasta and rice	-.21	**0.34**
Potatoes and tubers	-.02	**0.40**
Poultry	-.03	0.06
Processed meat	-.22	0.20
Pulses	0.19	0.02
Soups and broths	**0.26**	0.22
Sugar and confectionery	-.08	0.12
Sweetened soft drinks	**-.28**	-.00
Unsweetened soft drinks	**0.25**	0.15
Vegetables	**0.47**	0.23
Whole grains	**0.38**	-.04

Figure S1. Flowchart of study population within the NutriNet-Santé cohort, 2009-2021, N=103,389.

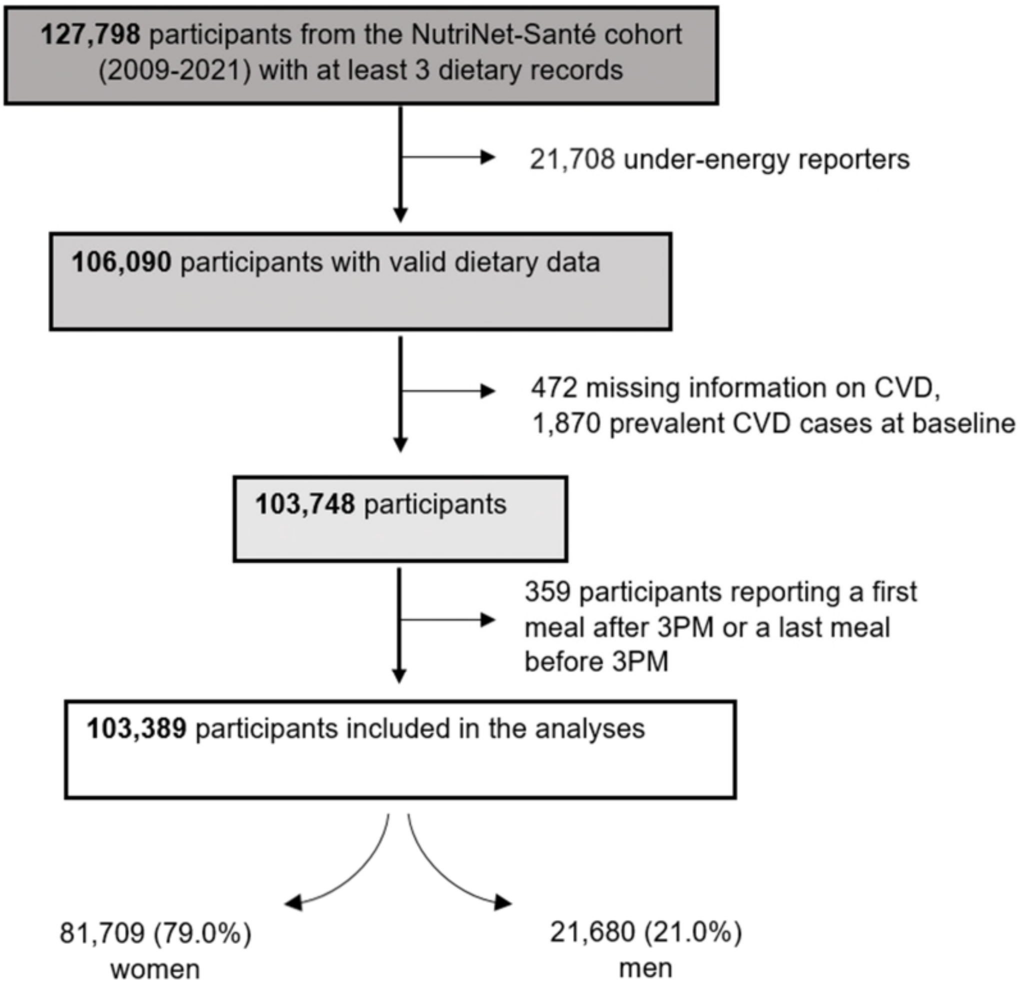

Figure S2. Correlations between meal timing, number of eating occasions and nighttime fasting duration in 103,389 participants from the NutriNet-Santé study (2009-2021). Source data are provided as a Source Data file.

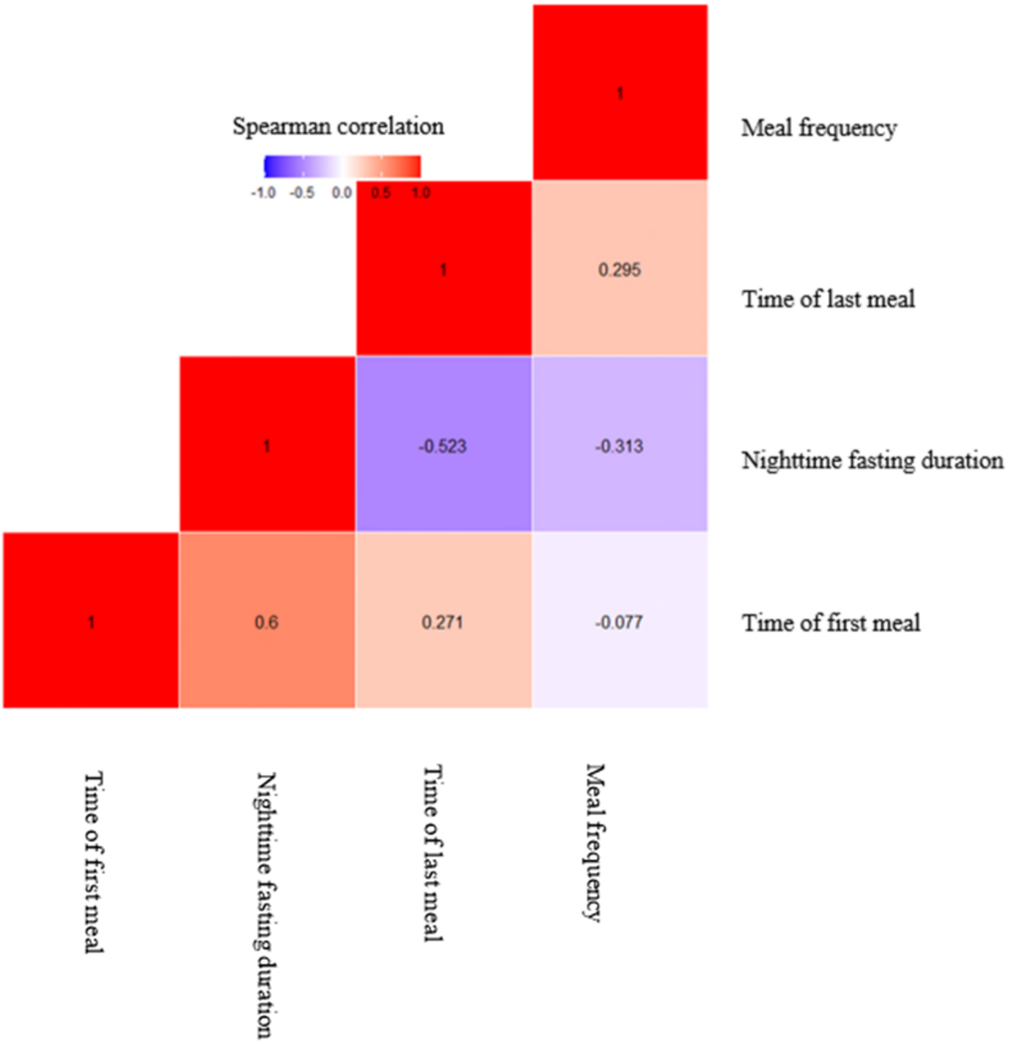

Figure S3. Distribution of meal timings, frequency and fasting duration in 103,389 participants from the NutriNet-Santé study (2009-2021). Mean point represented with a yellow dot. Source data are provided as a Source Data file.

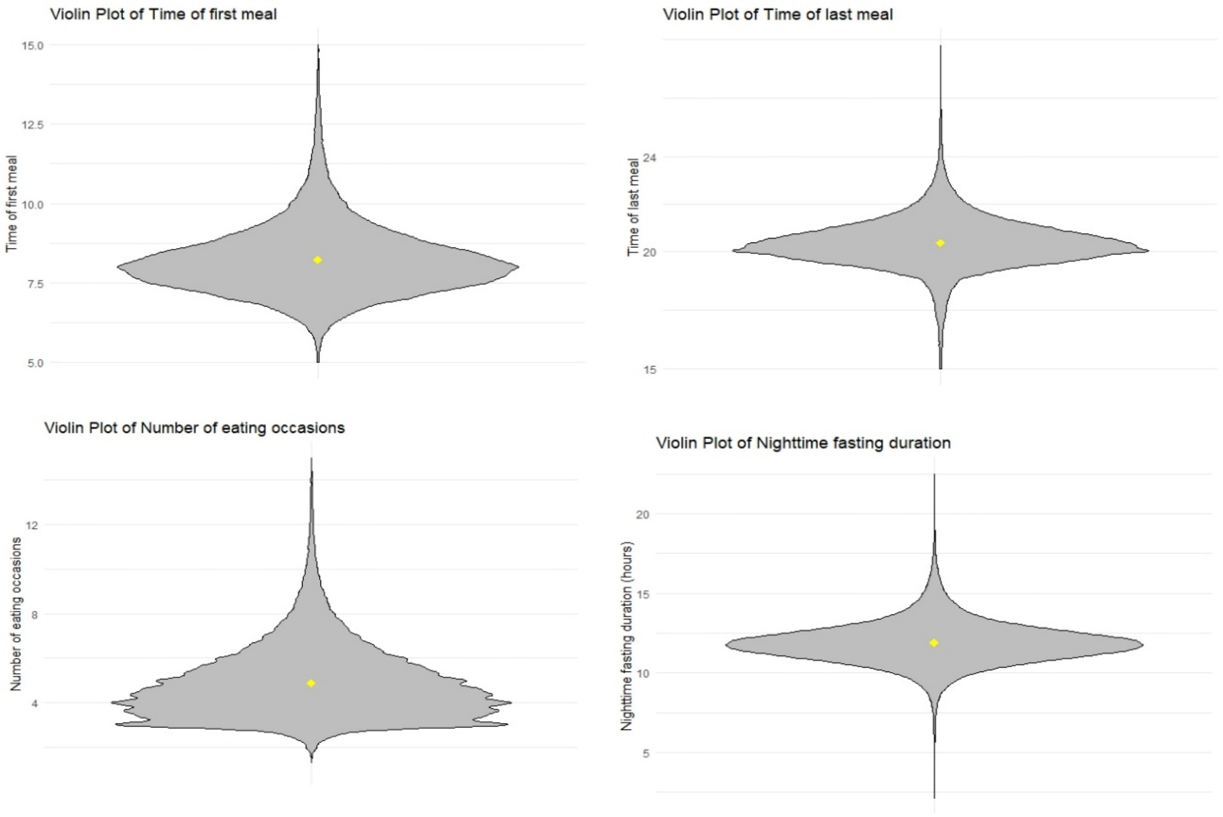

Figure S4. Linearity of the associations of meal timing and number of eating occasions with risk of overall cardiovascular diseases (first row, figures a,b,c), cerebrovascular diseases (second row, figures d,e,f) and coronary heart diseases (third row, figures g,h,i) (N=103,389). P-values for non linearity are indicated below each individual graph. CVD = cardiovascular disease; CVA = cerebrovascular disease; CHD = coronary heart disease. Source data are provided as a Source Data file.

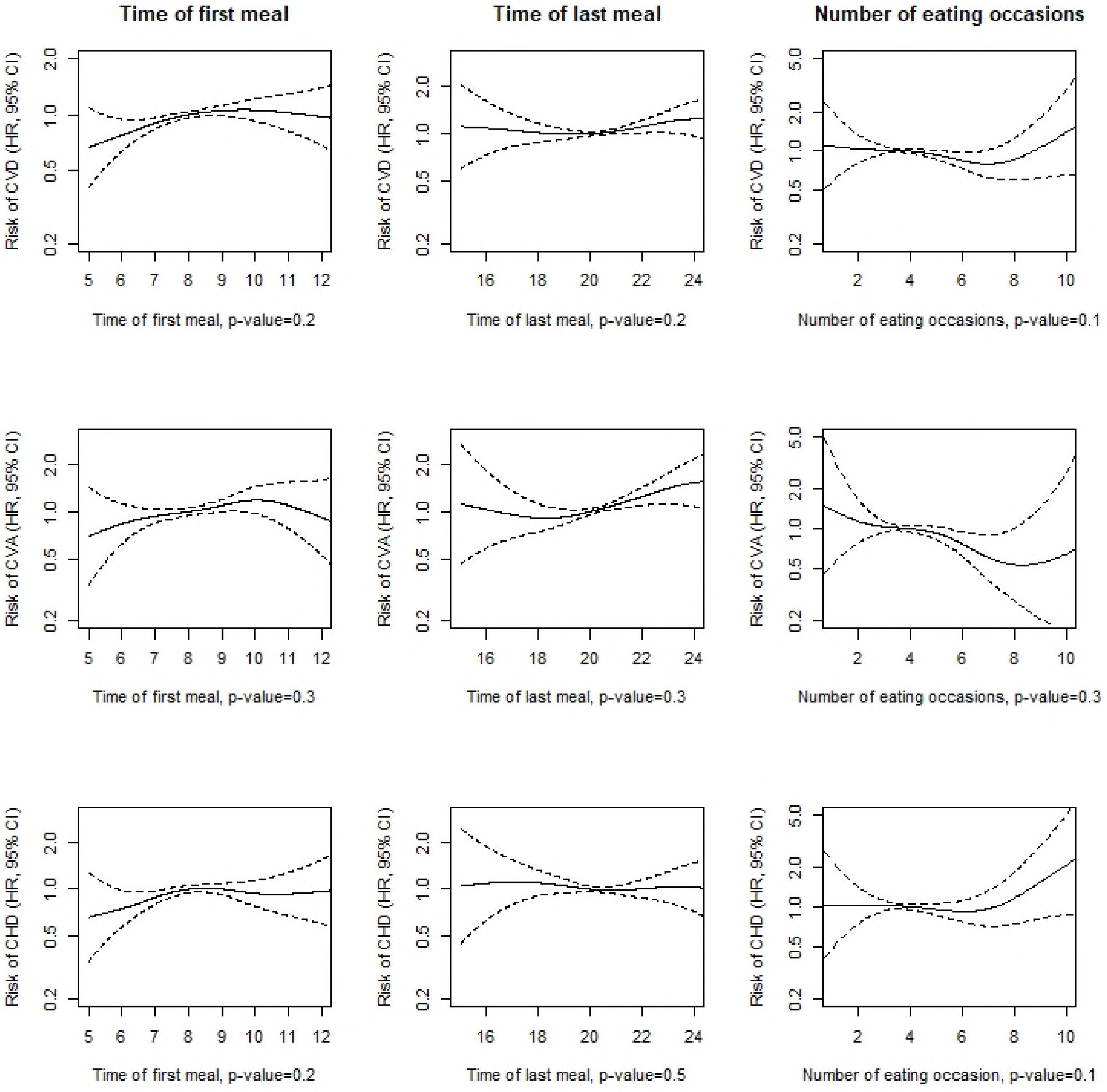

Figure S5. Schoenfled residuals in main models (N=103,389). Graphs correspond to models of overall cardiovascular disease (first row), cerebrovascular diseases (second row) and coronary heart diseases (third row) respectively. Source data are provided as a Source Data file.

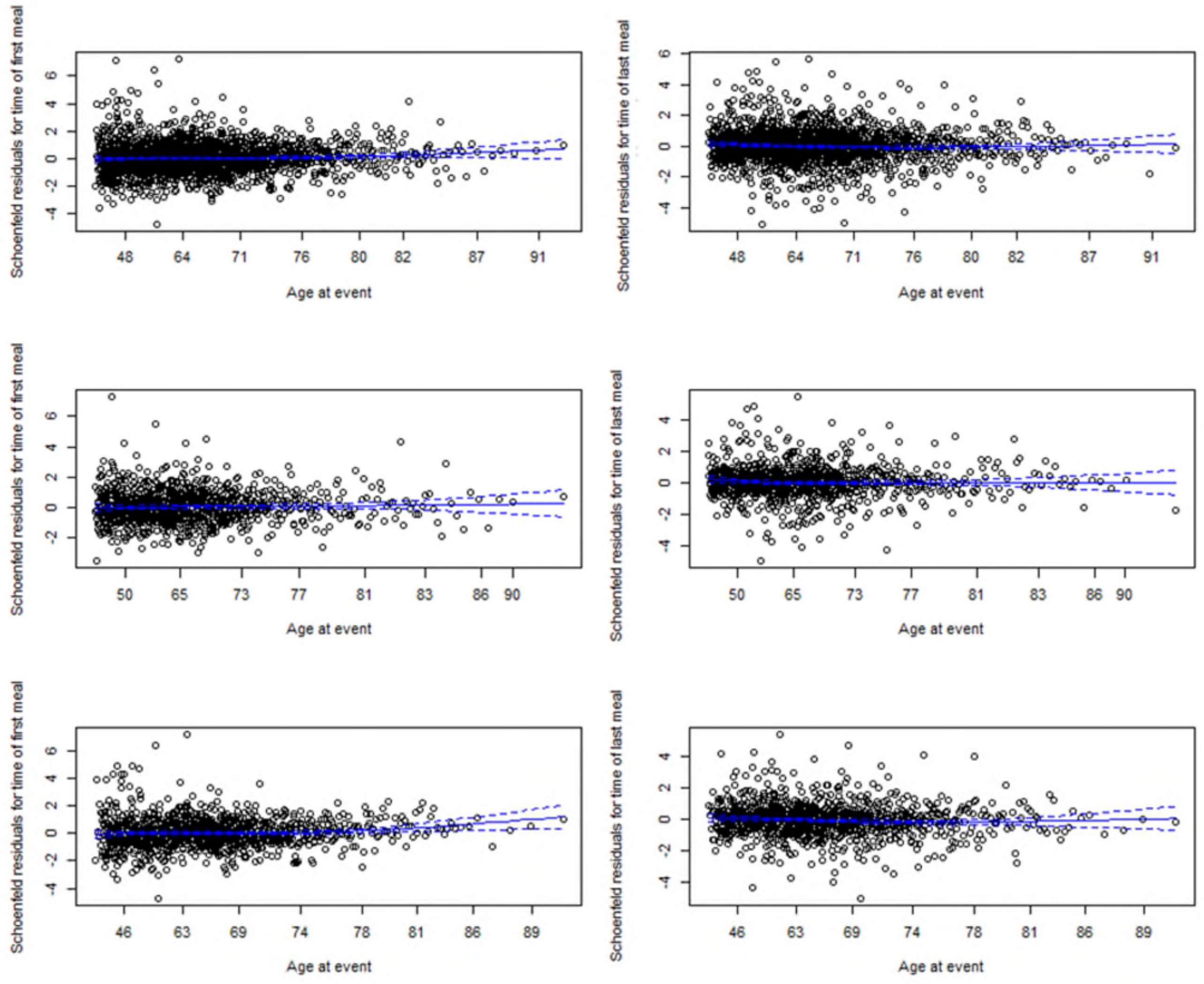

Figure S6. Generalized additive model examining the association of time interval between last meal of the day and bedtime with overall CVD risk (N=103,389). Source data are provided as a Source Data file.

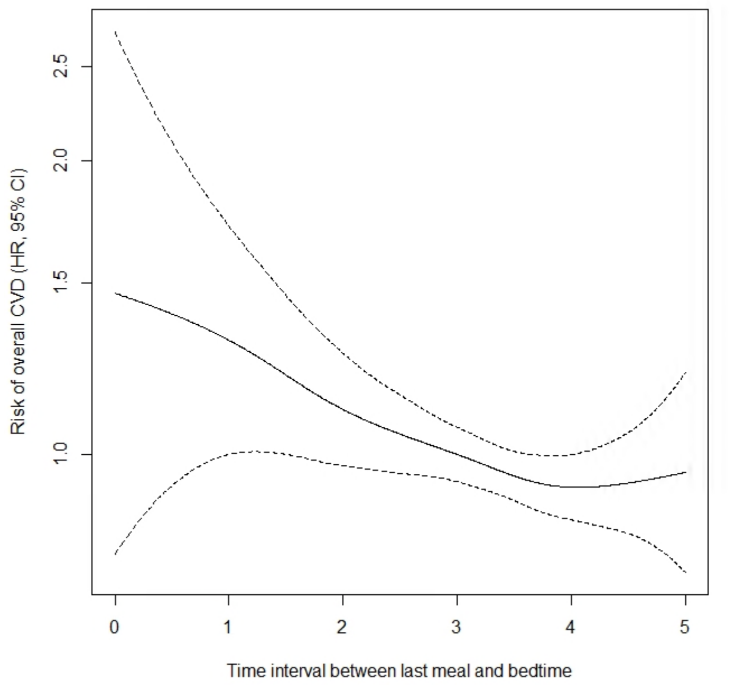

Table S1. Associations of meal timing and cardiovascular diseases in 103,389 participants from the NutriNet-Santé study (2009-2021) – sensitivity analyses

		Overall cardiovascular diseases			Cerebrovascular diseases‡			Coronary heart diseases §		
		N cases / non-cases	HR (95 % CI)	P-val	N cases / non-cases	HR (95 % CI)	P-val	N cases / non-cases	HR (95 % CI)	P-val
**Model 0**	Time of first meal (1h incr.)	2,036 / 101,353	1.06 (1.01 – 1.12)	0.02	988 / 102,401	1.06 (0.98 – 1.14)	0.1	1,071 / 102,318	1.05 (0.98 – 1.13)	0.1
	Time of last meal (1h incr.)		1.02 (0.98 – 1.07)	0.4		1.08 (1.01 – 1.15)	0.02		0.97 (0.92 – 1.05)	0.4
**Model 1**	Time of first meal	2,036 / 101,353	1.06 (1.01 – 1.11)	0.02	902 / 98,026	1.05 (0.98 – 1.13)	0.2	1,010 / 97,918	1.05 (0.98 – 1.12)	0.2
	Time of last meal		1.02 (0.97 – 1.07)	0.4		1.08 (1.01 – 1.15)	0.02		0.97 (0.91 – 1.04)	0.4
**Model 2**	Time of first meal	2,036 / 101,353	1.06 (1.00 – 1.11)	0.03	988 / 102,401	1.05 (0.97 – 1.13)	0.2	1,071 / 102,318	1.05 (0.98 – 1.13)	0.2
	Time of last meal		1.04 (0.99 – 1.09)	0.09		1.10 (1.03 – 1.17)	<0.01		0.98 (0.92 – 1.04)	0.6
**Model 3**	Time of first meal	1,999 / 98,267	1.06 (1.01 – 1.12)	0.02	964 / 99,302	1.06 (0.98 – 1.14)	0.1	1,058 / 99,302	1.05 (0.98 – 1.13)	0.1
	Time of last meal		1.02 (0.98 – 1.07)	0.4		1.08 (1.01 – 1.15)	0.02		0.97 (0.92 – 1.04)	0.4
**Model 4**	Time of first meal	2,014 / 98,842	1.06 (1.01 – 1.11)	0.02	977 / 99,879	1.06 (0.99 – 1.14)	0.1	1,060 / 99,796	1.04 (0.97 – 1.12)	0.2
	Time of last meal		1.02 (0.98 – 1.07)	0.3		1.08 (1.01 – 1.15)	0.02		0.98 (0.92 – 1.04)	0.5
**Model 5**	Time of first meal	1,611 / 101,349	1.06 (1.00 – 1.12)	0.05	809 / 102,400	1.05 (0.96 – 1.13)	0.3	807 / 102,315	1.07 (0.99 – 1.16)	0.1
	Time of last meal		1.03 (0.98 – 1.08)	0.2		1.09 (1.02 – 1.17)	0.01		0.97 (0.90 – 1.04)	0.4
**Model 6**	Time of first meal	2,036 / 101,353	1.06 (1.01 – 1.12)	0.01	988 / 102,401	1.06 (0.98 – 1.14)	0.1	1,071 / 102,318	1.05 (0.98 – 1.13)	0.1
	Time of last meal		1.02 (0.97 – 1.07)	0.4		1.08 (1.01 – 1.15)	0.02		0.97 (0.91 – 1.04)	0.4
**Model 7**	Time of first meal	1,532 / 44,436	1.08 (1.02 – 1.15)	0.01	759 / 45,209	1.07 (0.98 – 1.17)	0.1	790 / 45,178	1.07 (0.98 – 1.17)	0.1
	Time of last meal		1.00 (0.95 – 1.06)	1.0		1.04 (0.96 – 1.12)	0.3		0.97 (0.90 – 1.04)	0.4
**Model 8**	Time of first meal	1,196 / 36,340	1.10 (1.02 – 1.18)	<0.01	580 / 36,956	1.09 (0.98 – 1.20)	0.1	654 / 36,882	1.10 (1.00 – 1.21)	0.05
	Time of last meal		0.98 (0.92 – 1.04)	0.5		1.03 (0.94 – 1.13)	0.4		0.93 (0.85 – 1.01)	0.1
**Model 9**	Time of first meal	1,077 / 33,824	1.13 (1.04 – 1.22)	< 0.01	524 / 34,377	1.11 (0.99 – 1.24)	0.08	562 / 34,339	1.13 (1.02 – 1.26)	0.02
	Time of last meal		0.96 (0.87 – 1.05)	0.4		1.02 (0.89 – 1.17)	0.8		0.91 (0.80 – 1.04)	0.1
**Model 10**	Time of first meal	1,161 / 35,310	1.07 (0.99 – 1.16)	0.07	562 / 35,909	1.09 (0.98 – 1.22)	0.1	609 / 35,862	1.05 (0.94 – 1.17)	0.4
	Time of last meal		0.98 (0.91 – 1.04)	0.5		1.04 (0.94 – 1.14)	0.4		0.92 (0.84 – 1.01)	0.07
**Model 11**	Time of first meal	1,196 / 36,324	1.10 (1.02 – 1.18)	< 0.01	580 / 36,940	1.09 (0.98 – 1.20)	0.1	627 / 36,893	1.10 (1.00 – 1.21)	0.05
	Time of last meal		0.98 (0.92 – 1.05)	0.6		1.03 (0.94 – 1.13)	0.5		0.93 (0.85 – 1.02)	0.1
**Model 12**	Time of first meal	2,052 / 101,696	1.06 (1.01 – 1.11)	0.01	998 / 102,750	1.06 (0.99 – 1.13)	0.07	1,085/ 102,663	1.05 (0.98 – 1.12)	0.1
	Time of last meal		1.01 (0.97 – 1.06)	0.6		1.09 (1.02 – 1.16)	<0.01		0.95 (0.90 – 1.01)	0.1
**Model 13**	Time of first meal	2,025 / 101,091	1.06 (1.01 – 1.12)	0.01	984 / 102,132	1.06 (0.97 – 1.14)	0.1	1,064 / 102,052	1.05 (0.98 – 1.13)	0.1
	Time of last meal		1.02 (0.97 – 1,07)	0.4		1.08 (1.01 – 1.15)	0.02		0.97 (0.91 – 1.03)	0.4
**Model 14**	Time of first meal	2,036 / 101,353	1.05 (1.00 – 1.10)	0.06	988 / 102,401	1.05 (0.97 – 1.12)	0.2	1,071 / 102,318	1.04 (0.97 – 1.11)	0.3
	Time of last meal		1.02 (0.97 – 1.06)	0.4		1.07 (1.01 – 1.15)	0.03		0.97 (0.91 – 1.03)	0.4
**Model 15**	Time of first meal	2,036 / 101,353	1.06 (1.01 – 1.12)	0.01	988 / 102,401	1.06 (0.99 – 1.14)	0.1	1,071 / 102,318	1.05 (0.98 – 1.13)	0.1
	Time of last meal		1.02 (0.97 – 1.07)	0.4		1.08 (1.01 – 1.15)	0.03		0.97 (0.92 – 1.04)	0.4
**Model 16**	Time of first meal	2,036 / 101,353	1.06 (1.01 – 1.12)	0.01	988 / 102,401	1.06 (0.98 – 1.14)	0.1	1,071 / 102,318	1.06 (0.99 – 1.13)	0.1
	Time of last meal		1.02 (0.97 – 1.07)	0.4		1.08 (1.01 – 1.15)	0.02		0.97 (0.92 – 1.03)	0.4
**Model 17**	Time of first meal	2,036 / 101,353	1.06 (1.01 – 1.12)	0.01	988 / 102,401	1.06 (0.99 – 1.14)	0.1	1,071 / 102,318	1.05 (0.98 – 1.13)	0.1
	Time of last meal		1.02 (0.97 – 1.07)	0.4		1.08 (1.01 – 1.15)	0.02		0.97 (0.92 – 1.04)	0.4
**Model 18**	Time of first meal	2,036 / 101,353	1.06 (1.01 – 1.12)	0.02	988 / 102,401	1.06 (0.98 – 1.14)	0.1	1,071 / 102,318	1.05 (0.98 – 1.13)	0.1

	Time of last meal		1.02 (0.97 – 1.07)	0.4		1.08 (1.01 – 1.15)	0.03		0.97 (0.91 – 1.03)	0.4
**Model 19**	Time of first meal	1,006 / 103,374	1.03 (0.95 – 1.11)	0.5						
	Time of last meal		0.97 (0.90 – 1.03)	0.3						

HR= Hazard ratio; N = Sample size; CI= Confidence Interval.
‡ Stroke and transient ischemic attack. § Myocardial infarction, acute coronary syndrome, angioplasty and angina pectoris.
Model 0. Multivariable Cox proportional hazard models adjusted for age (timescale), sex (women, men), educational level (less than high school degree, <2 years after high school degree, ≥2 years after high school degree), monthly income per unit of consumption (less than 900€, 900-1,200€, 1,200-1,800€, 1,800-2,300€, 2,300-3,700€, more than 3,700€, don't want to answer), BMI at baseline (continuous, kg/m2), family history of CVDs (no, yes), alcohol consumption (Non-consumers (0g/day), low consumers (0.1 – 4.9 g/day), moderate consumers (5.0 – 14.9 g/day), high consumers (15.0 -29.9 g/day) and very high consumers (>30.0 g/day)), episodes of binge drinking (None, one, more than one) alcohol intake (continuous, kcal/day), daily energy intake excluding alcohol (continuous, kcal/day), healthy and Western dietary patterns derived by factorial analysis (continuous), smoking (current regular (1 cigarette or more per day), current occasional, current, former, never), number of pack years (continuous, defined as the number of packs of cigarettes smoked per day by the number of years of smoking), physical activity (low, moderate, high), number of dietary records (continuous) and number of eating occasions (continuous). Time of first and last meal were mutually adjusted.
Model 1. Without healthy diet pattern, western diet pattern and adding daily consumption of saturated fatty acids, sodium, sugar, red and processed meat, sugary drinks, fruits and vegetables, nuts, whole grain (g/d, continuous) and ultra-processed food (daily weight proportion, continuous).
Model 2. Adding region of participants (Bassin parisien, Centre Est France, Est France, Méditerranée, Nord France, Ouest France, Ille de France) and profession (Unemployed; Student; Self-employed, farmer; Employed, manual worker; Intermediate professions; Managerial staff; Intellectual profession; Retired)
Model 3. Adding percentage of weight change during follow-up calculated as the percentage of weight change from baseline to end of study and divided by number of years of follow-up.
Model 4. Adding eating jet lag. This variable was categorized as an "advance" in meal timing of non-working compared to working days if values were below -1, "maintenance" if values were between -1 and 1 and "delay" if values were higher than 1.
Model 5. Excluding participants diagnosed during the first 2 years of follow-up.
Model 6. Adjusting for prevalent cases of type 1 and 2 diabetes, hypercholesterolemia, hypergliceridemia and hypertension at baseline.
Model 7. Analyses restricted to participants with sleep data (N= 45,968).
Model 8. Analyses restricted to participants with sleep data, reporting a bedtime between 8AM and 6PM (N=37,536).
Model 9. As model 8 and adjusting for sleep duration (hrs. / 24h, continous) and time interval between last meal and betime (N= 34,901). We calculated the time interval between bedtime and last meal, after excluding participants (7%) having reported a time of last meal (assessed at baseline) posterior to their bedtime (assessed in 2014), N= 34,901.
Model 10. As model 8 and adjusting for chronotype (morning, intermediate, evening).
Model 11. As model 8 and adjusting for number of awakenings and for sleep apnea, N= 37,520.
Model 12. As model 0 but without excluding participants with extreme eating patterns (having a first meal after 3PM or a last eating occasion before 3PM), N=103,748.
Model 13. As model 0 and adding marital status (Married; Couple; Divorced or separated; Widower; Single) and number of children.
Model 14. As model 0 and adding number of medications.
Model 15. As model 0 and adding season when the physical activity questionnaire was completed.
Model 16. As model 0 and adding unusual dietary reporting.
Model 17. As model 0 and adding restrictive diets.
Model 18. As model 0 and adding season of first dietary record.
Model 19. Model 0 but in association with basal cell carcinoma (falsification endpoint).
Source data are provided as a Source Data file.

Table S2. Association of meal timing and number of eating occasions with risk of cardiovascular diseases in the NutriNet-santé cohort, 2009-2021, N= 103,368

	N cases / non-cases	HR (95% CI) 1	p-val	N cases / non-cases	HR (95% CI) 1	p-val	N cases / non-cases	HR (95% CI) 1	p-val
	Excluding prevalent cases of obesity (N=94,366)			Excluding prevalent cases of diabetes (N=101,415)			Excluding prevalent cases of apnea (N=45,645)		
**Overall cardiovascular diseases**									
Time of first meal (1h incr.)	1,866 / 92,500	1.07 (1.01 – 1.13)	0.01	1,879 / 99,536	1.06 (1.00 – 1.11)	0.04	1,520 / 44,125	1.07 (1.01 – 1.14)	0.03
Time of last meal (1h incr.)	1,866 / 92,500	1.01 (0.97 – 1.06)	0.6	1,879 / 99,536	1.01 (0.97 – 1.06)	0.5	1,520 / 44,125	1.00 (0.95 – 1.06)	0.9
Number of eating occasions (1 occasion incr.)	1,866 / 92,500	0.99 (0.96 – 1.02)	0.4	1,879 / 99,536	0.99 (0.96 – 1.01)	0.3	1,520 / 44,125	0.99 (0.95 – 1.02)	0.5
**Cerebrovascular diseases‡**									
Time of first meal (1h incr.)	902 / 93,464	1.06 (0.99 – 1.15)	0.1	938 / 100,477	1.04 (0.97 – 1.12)	0.3	752 / 44,893	1.07 (0.98 – 1.17)	0.1
Time of last meal (1h incr.)	902 / 93,464	1.07 (1.00 – 1.15)	0.04	938 / 100,477	1.08 (1.01 – 1.15)	0.02	752 / 44,893	1.04 (0.96 – 1.12)	0.4
Number of eating occasions (1 occasion incr.)	902 / 93,464	0.97 (0.92 – 1.01)	0.1	938 / 100,477	0.96 (0.92 – 1.01)	0.09	752 / 44,893	0.97 (0.93 – 1.02)	0.3
**Coronary heart diseases §**									
Time of first meal (1h incr.)	986 / 93,380	1.06 (0.98 – 1.14)	0.1	964 / 100,451	1.05 (0.98 – 1.13)	0.1	785 / 44,860	1.06 (0.97 – 1.15)	0.2
Time of last meal (1h incr.)	986 / 93,380	0.97 (0.91 – 1.03)	0.3	964 / 100,451	0.96 (0.90 – 1.02)	0.2	785 / 44,860	0.97 (0.90 – 1.05)	0.5
Number of eating occasions (1 occasion incr.)	986 / 93,380	1.01 (0.97 – 1.05)	0.7	964 / 100,451	1.01 (0.97 – 1.05)	0.7	785 / 44,860	1.00 (0.96 – 1.05)	0.9

HR= Hazard ratio; N= Sample size; CI= Confidence Interval.

‡ Stroke and transient ischemic attack. § Myocardial infarction, acute coronary syndrome, angioplasty and angina pectoris.

1. Multivariable Cox proportional hazard models adjusted for age (timescale), sex (women, men), educational level (less than high school degree, <2 years after high school degree, ≥2 years after high school degree), monthly income per unit of consumption (less than 900€, 900-1,200€, 1,200-1,800€, 1,800-2,300€, 2,300-3,700€, more than 3,700€, don't want to answer), BMI at baseline (continuous, kg/m2), family history of CVDs (no, yes), alcohol consumption (Non-consumers (0g/day), low consumers (0.1 – 4.9 g/day), moderate consumers (5.0 – 14.9 g/day), high consumers (15.0 -29.9 g/day) and very high consumers (>30.0 g/day)), episodes of binge drinking (None, one, more than one) , daily energy intake excluding alcohol (continuous, kcal/day), healthy and Western dietary patterns derived by factorial analysis (continuous), smoking (current regular (1 cigarette or more per day), current occasional, former, never), number of pack years (continuous, defined as the number of packs of cigarettes smoked per day by the number of years of smoking), physical activity (low, moderate, high) and number of dietary records (continuous). Time of first and last meal and number of eating occasions were mutually adjusted. Source data are provided as a Source Data file.

86. Black AE. The sensitivity and specificity of the Goldberg cut-off for EI: BMR for identifying diet reports of poor validity. Eur J Clin Nutr. 2000;54:395–404.

87. Goldberg GR, Black AE, Jebb SA, Cole TJ, Murgatroyd PR, Coward WA, et al. Critical evaluation of energy intake data using fundamental principles of energy physiology: 1. Derivation of cut-off limits to identify under-recording. Eur J Clin Nutr. 1991;45(12):569–81.

88. Schofield WN. Predicting basal metabolic rate, new standards and review of previous work. Hum Nutr Clin Nutr. 1985;39 Suppl 1:5–41.

89. Anses. Etude Individuelle Nationale des Consommations Alimentaires 3 (INCA 3) [Internet]. [cited 2022 May 16]. Available from: https://www.anses.fr/fr/content/inca-3-evolution-des-habitudes-et-modes-de-consommation-de-nouveaux-enjeux-en-mati%C3%A8re-de
