# Supplementary material for: Dietary circadian rhythms and cardiovascular disease risk in the prospective NutriNet-Santé cohort
Source: Nat Commun. 2023 Dec 14;14:7899. doi: 10.1038/s41467-023-43444-3 (PMC10721609; doi:10.1038/s41467-023-43444-3)
Supplement: Supplementary file 2 — Reporting Summary [file 41467_2023_43444_MOESM2_ESM.pdf]

nature portfolio

Corresponding author(s): Bernard Srour

Last updated by author(s): Oct 5, 2023

Reporting Summary

Nature Portfolio wishes to improve the reproducibility of the work that we publish. This form provides structure for consistency and transparency in reporting. For further information on Nature Portfolio policies, see our Editorial Policies and the Editorial Policy Checklist.

Statistics

For all statistical analyses, confirm that the following items are present in the figure legend, table legend, main text, or Methods section.

n/a	Confirmed	
☐	☒	The exact sample size (*n*) for each experimental group/condition, given as a discrete number and unit of measurement
☐	☒	A statement on whether measurements were taken from distinct samples or whether the same sample was measured repeatedly
☐	☒	The statistical test(s) used AND whether they are one- or two-sided<br>*Only common tests should be described solely by name; describe more complex techniques in the Methods section.*
☐	☒	A description of all covariates tested
☐	☒	A description of any assumptions or corrections, such as tests of normality and adjustment for multiple comparisons
☐	☒	A full description of the statistical parameters including central tendency (e.g. means) or other basic estimates (e.g. regression coefficient) AND variation (e.g. standard deviation) or associated estimates of uncertainty (e.g. confidence intervals)
☐	☒	For null hypothesis testing, the test statistic (e.g. *F*, *t*, *r*) with confidence intervals, effect sizes, degrees of freedom and *P* value noted<br>*Give P values as exact values whenever suitable.*
☒	☐	For Bayesian analysis, information on the choice of priors and Markov chain Monte Carlo settings
☒	☐	For hierarchical and complex designs, identification of the appropriate level for tests and full reporting of outcomes
☒	☐	Estimates of effect sizes (e.g. Cohen's *d*, Pearson's *r*), indicating how they were calculated

Our web collection on statistics for biologists contains articles on many of the points above.

Software and code

Policy information about availability of computer code

Data collection	No software was used for data collection.
Data analysis	R version 4.1.3 (The R Foundation for Statistical Computing Platform) was used for these analyses.

For manuscripts utilizing custom algorithms or software that are central to the research but not yet described in published literature, software must be made available to editors and reviewers. We strongly encourage code deposition in a community repository (e.g. GitHub). See the Nature Portfolio guidelines for submitting code & software for further information.

Data

Policy information about availability of data

All manuscripts must include a data availability statement. This statement should provide the following information, where applicable:

- Accession codes, unique identifiers, or web links for publicly available datasets
- A description of any restrictions on data availability
- For clinical datasets or third party data, please ensure that the statement adheres to our policy

Data described in the manuscript are protected and are not available due to data privacy laws. It will be made available upon request pending application and approval. Researchers from public institutions can submit a collaboration request including information on the institution and a brief description of the project to collaboration@etude-nutrinet-sante.fr. All requests will be reviewed by the steering committee of the NutriNet-Santé study. If the collaboration request is accepted, a data access agreement will be necessary and appropriate authorizations from the competent administrative authorities may be needed. In accordance with existing regulations, no personal identification data will be accessible. The NutriNet-Santé food composition database is available in the book "Table de

composition des aliments, Etude NutriNet-Santé, Editions Inserm – Economia", ISBN-10 : 2717865373 ISBN-13 : 978-2717865370.

Research involving human participants, their data, or biological material

Policy information about studies with human participants or human data. See also policy information about sex, gender (identity/presentation), and sexual orientation and race, ethnicity and racism.

Reporting on sex and gender	We have carefully used the term sex to avoid confusion with gender. Sex was determined based on self-reporting and we present sex-based analyses.
Reporting on race, ethnicity, or other socially relevant groupings	We adjusted our principal models for education (no high-school degree, less than 2 years of education after high-school, 2 years of more of high school education) and monthly income per unit of consumption (less than 900€, 900-1,200€, 1,200-1,800€, 1,800-2,300€, 2,300-3,700€, more than 3,700€, don't want to answer) as indicators of socioeconomic status.<br><br>In sensitivity analyses, we explored further adjustment of our models for profession, marital status and number of children.<br><br>All these variables were self-reported by the study participants in the baseline questionnaire.
Population characteristics	The present study included a total of 103,389 participants (79% women) with a mean baseline age of 42.6 years (14.5 SD). Participants completed on average 5.7 dietary records (SD 3.0) with a maximum of 15 records.
Recruitment	Participant are recruited from the general population of French adults (aged >18 years) through vast multimedia campaigns. To enroll, participants are required to create a personal account on the NutriNet-Santé web-based platform (https://etude-nutrinet-sante.fr/).
Ethics oversight	NutriNet-Santé is conducted according to the Declaration of Helsinki guidelines and was approved by the institutional review board of the French Institute for Health and Medical Research (IRB Inserm n 0000388FWA00005831) and the Commission Nationale de l'Informatique et des Libertés (CNIL n 908450/n 909216).

Note that full information on the approval of the study protocol must also be provided in the manuscript.

Field-specific reporting

Please select the one below that is the best fit for your research. If you are not sure, read the appropriate sections before making your selection.

[x] Life sciences   [ ] Behavioural & social sciences   [ ] Ecological, evolutionary & environmental sciences

For a reference copy of the document with all sections, see nature.com/documents/nr-reporting-summary-flat.pdf

Life sciences study design

All studies must disclose on these points even when the disclosure is negative.

Sample size	The present study included a total of 103,389 participants. No previous calculation for sample size were performed. We used data from all participants having at least 3 dietary records with no prevalent cardiovascular disease at baseline. No a priori sample size calculation was needed for this analysis.
Data exclusions	We excluded 21,708 under-energy reporters, 472 participants with missing information on CVD, 1,870 prevalent CVD cases at baseline. We used basal metabolic rate and the Goldberg cut-off method 38 to identify and exclude energy under-reporters. Finally, we excluded 359 participants reporting a first meal after 3PM and a last meal before 3PM, since they corresponded to very disrupted circadian behaviours (e.g. night shift workers).
Replication	N/A. This is an exploratory etiological analysis and not a predictive model. No replication or cross-validation is needed within the cohort.
Randomization	N/A (observational)
Blinding	N/A (observational)

Reporting for specific materials, systems and methods

We require information from authors about some types of materials, experimental systems and methods used in many studies. Here, indicate whether each material, system or method listed is relevant to your study. If you are not sure if a list item applies to your research, read the appropriate section before selecting a response.

Materials & experimental systems

n/a	Involved in the study
☒	☐ Antibodies
☒	☐ Eukaryotic cell lines
☒	☐ Palaeontology and archaeology
☒	☐ Animals and other organisms
☒	☐ Clinical data
☒	☐ Dual use research of concern
☒	☐ Plants

Methods

n/a	Involved in the study
☒	☐ ChIP-seq
☒	☐ Flow cytometry
☒	☐ MRI-based neuroimaging
